# Primary Small Cell Neuroendocrine Tumour of Gallbladder Presenting as Pyrexia of Unknown Origin

**DOI:** 10.7759/cureus.15874

**Published:** 2021-06-23

**Authors:** Arkadeep Dhali, Sukanta Ray, Gopal Krishna Dhali

**Affiliations:** 1 Department of Gastrointestinal Surgery, School of Digestive and Liver Diseases, Institute of Postgraduate Medical Education and Research, Kolkata, IND; 2 Department of Gastroenterology, School of Digestive and Liver Diseases, Institute of Postgraduate Medical Education and Research, Kolkata, IND

**Keywords:** neuroendocrine tumor, gallbladder malignancy, small cell tumour, radical cholecystectomy, pyrexia of unknown origin

## Abstract

Herein, we report a case of primary small cell neuroendocrine tumor (NET) of the gallbladder in a 45-year-old female who presented with complaints of fever accompanied by abdominal pain on the right upper quadrant for one month. Contrast-enhanced computed tomography abdomen showed a large gallbladder mass. It was infiltrating the segments IVa, IVb, and V of the liver. Ultrasound-guided fine-needle-aspiration-cytology was performed. Based on preoperative pathological and immunohistochemical investigations, it was diagnosed to be a primary small cell NET of the gallbladder. The patient underwent radical cholecystectomy after three cycles of cisplatin-based neoadjuvant chemotherapy. She had an uneventful recovery and received adjuvant chemo-radiotherapy. The patient was well at the 18-month follow-up.

## Introduction

The primary small cell variety of neuroendocrine tumor (NET) of the gall bladder is a rare subtype of NET with approximately 40 cases reported in the English literature [1]. It accounts for 2.1% of all cases of gallbladder malignancy and was first described by Oberndorfer as carcinoid in 1907 [2]. These are heterogeneous in nature and originate from neuroendocrine cells and Kulchitsky cells [3]. They can either be secretory in nature which produces biologically active peptides or they can be nonsecretory having symptoms developing due to mass effect [4]. NETs arise mostly from the gastrointestinal tract (73.7%) or the bronchopulmonary system (25.1%) [5]. According to a population-based study of Kanakala et al., it was suggested that incidence NET of the gallbladder was as low as 0.2-1% [6]. Generally, gallbladder carcinomas (adenocarcinoma) are known to have a very poor prognosis with a five-year survival rate of approximately 5% whereas the survival rate for NET is even lower due to its late stage of presentation and highly malignant nature [7]. Most of the patients are managed with a combination of chemotherapy, radiotherapy, and surgical intervention. They are generally seen in elderly females with cholelithiasis with a significant risk factor being cigarette smoking [8]. Herein, we report a case of NET gallbladder that was successfully managed at our institution. 

## Case presentation

A 45-year-old lady presented with a one-month history of on and off fever with occasional chills and rigors relieved by taking oral paracetamol. She also complained of dull aching abdominal pain in the right upper quadrant which had no relation with food intake and not relieved by passage of stool and flatus. There was no history of cough, breathlessness, jaundice, weight loss, and easy fatigability. There were no urinary symptoms. Physical examination revealed significant pallor. Abdominal examination revealed a 4 x 2 cm gallbladder mass.

**Figure 1:**
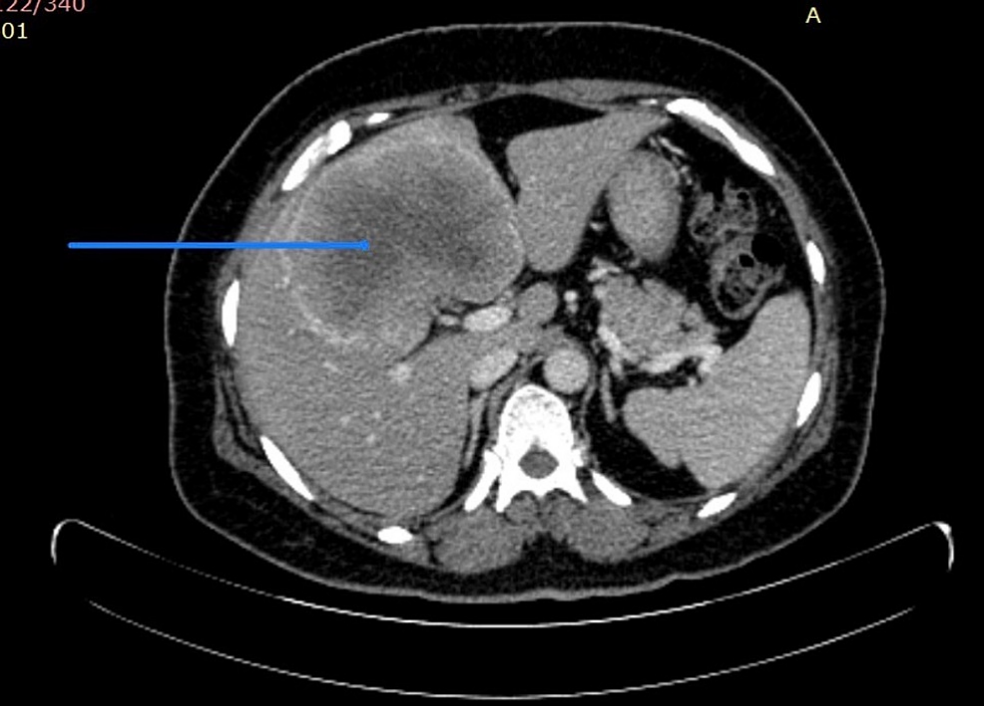
CECT showing an ill-defined heterogeneously enhancing wall thickening in the fundus of the gallbladder extending into the segment IV a, IV b, and V of the liver (blue arrow)

**Figure 2:**
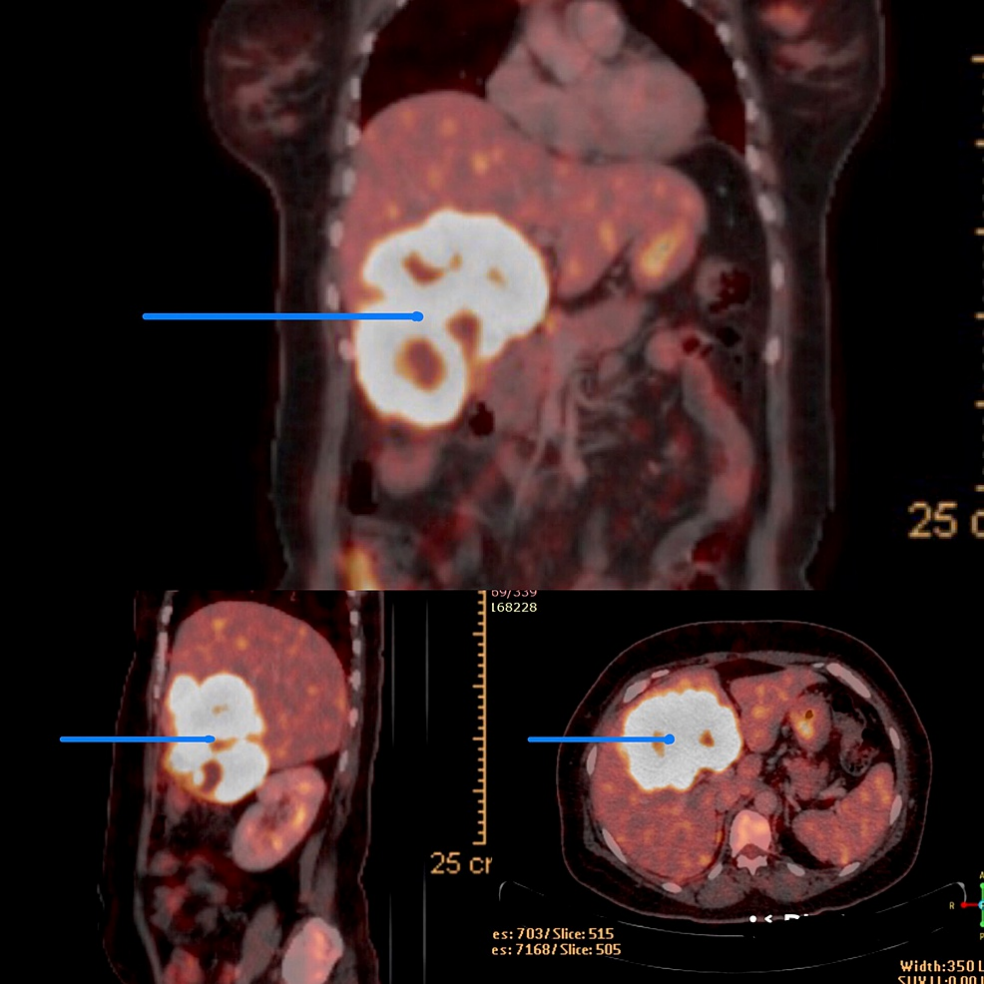
PET-CT scan showing 18-FDG wall thickening involving the gallbladder fundus with direct infiltration of the liver in the form of a large heterogeneously enhancing, partially necrotic intense 18-FDG avid with mass lesion in segment IV and V measuring approximately 9.6 x 8.4 x 10.1 cm, SUV max of 15.6 (blue arrows)

**Figure 3:**
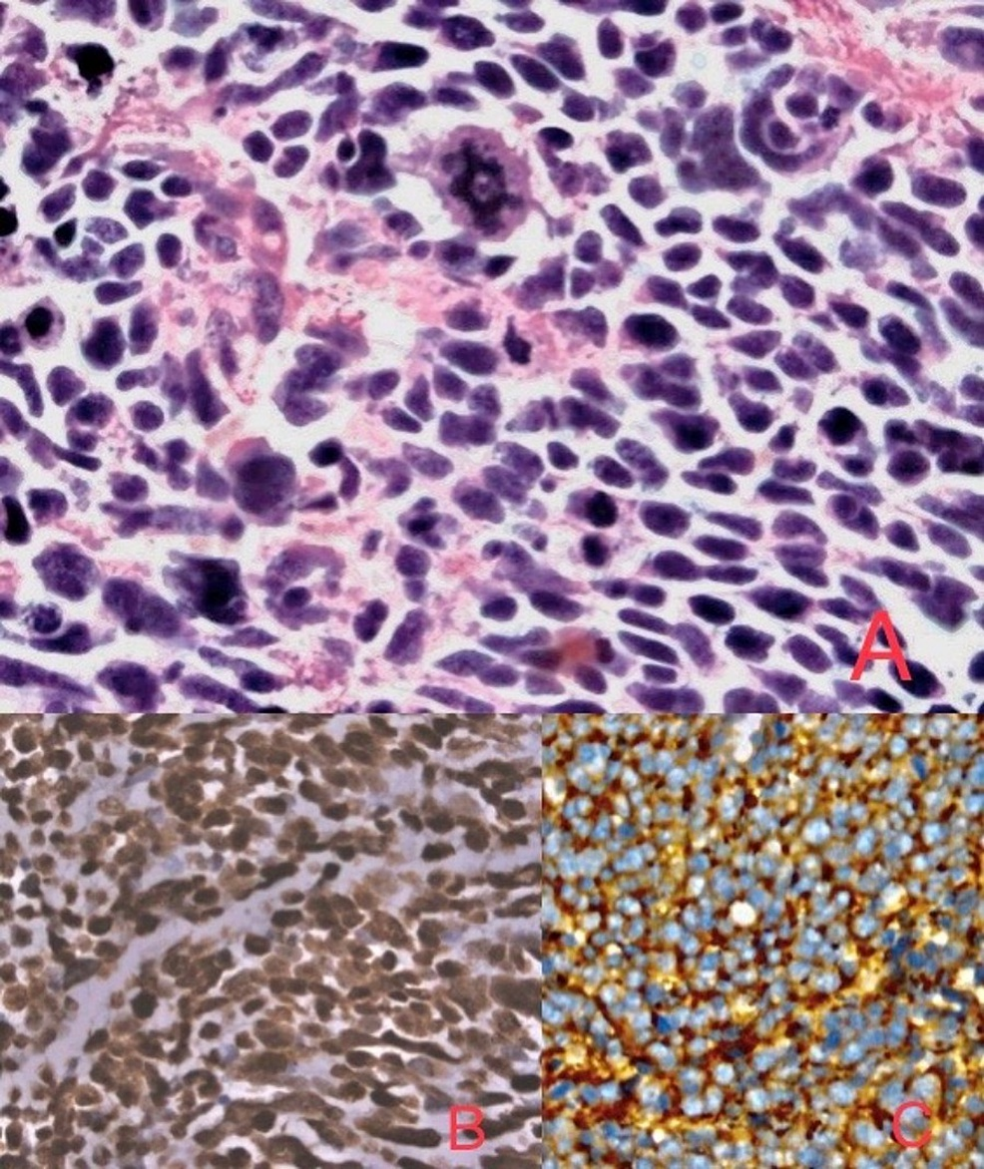
A: H&E 400X nuclear moldings with many abnormal mitotic figures, apoptotic cells; B: IHC 400X: strongly diffuse positive for chromogranin A; C: IHC- positive for synaptophysin

Preliminary laboratory investigations showed hemoglobin of 6.5g/dl, WBC count of 12400 cells/cu.mm, and platelets of 2.1 lakhs. Serum bilirubin, transaminases, and alkaline phosphatase levels were within normal limits. CA-19-9 and CEA were normal. Initial ultrasonography (USG) abdomen showed a space-occupying lesion (SOL) in the right lobe of the liver which was continuous with the multiseptate cystic SOL in the gallbladder. Chest X-ray was normal. Contrast-enhanced computed tomography (CECT) showed an ill-defined heterogeneously enhancing wall thickening in the fundus of gall bladder extending into the segment IVa, IVb, and V of the liver (Figure 1). There was enlargement of the periportal lymph nodes. Further evaluation with PET-CT scans showed intense 18-fluorodeoxyglucose (FDG) wall thickening involving the gallbladder fundus with direct infiltration of the liver in the form of a large heterogeneously enhancing, partially necrotic intense 18-FDG avid with a mass lesion in segment IV and V measuring approximately 9.6 x 8.4 x 10.1 cm, SUV max of 15.6 (Figure 2). There was no evidence of calculi, low tracer avid was noted in the adjacent portal lymph nodes. Findings were likely to represent stage-III carcinoma of gall bladder with direct infiltration of the liver. As the tumor was locally advanced, a USG-guided fine-needle-aspiration-cytology was performed which was inconclusive for malignancy. Further evaluation with USG guided core-needle biopsy revealed an infiltrating neoplasm composed of tumor cells arranged in nests and occasional glandular patterns. The cells showed elongated hyperchromatic nuclei with scant cytoplasm. For better characterization of malignancy an immunohistochemical (IHC) panel was performed (positive for CK7, CK20, CK19, synaptophysin, chromogranin, Ki67- 90%) which was suggestive of NET (Figure 3).

**Figure 4:**
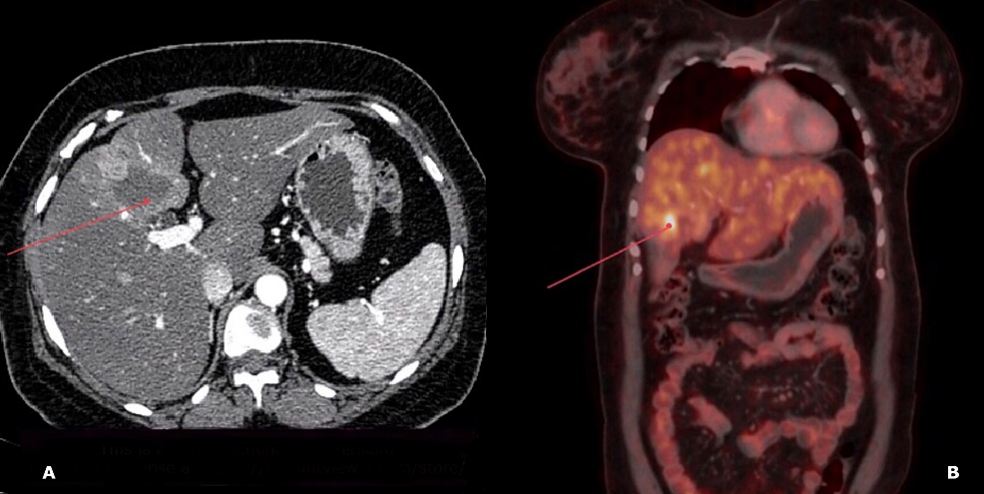
CECT(A) and PET-CT(B) images showing shrinkage of the size of tumor post neoadjuvant chemotherapy (red arrows)

**Figure 5:**
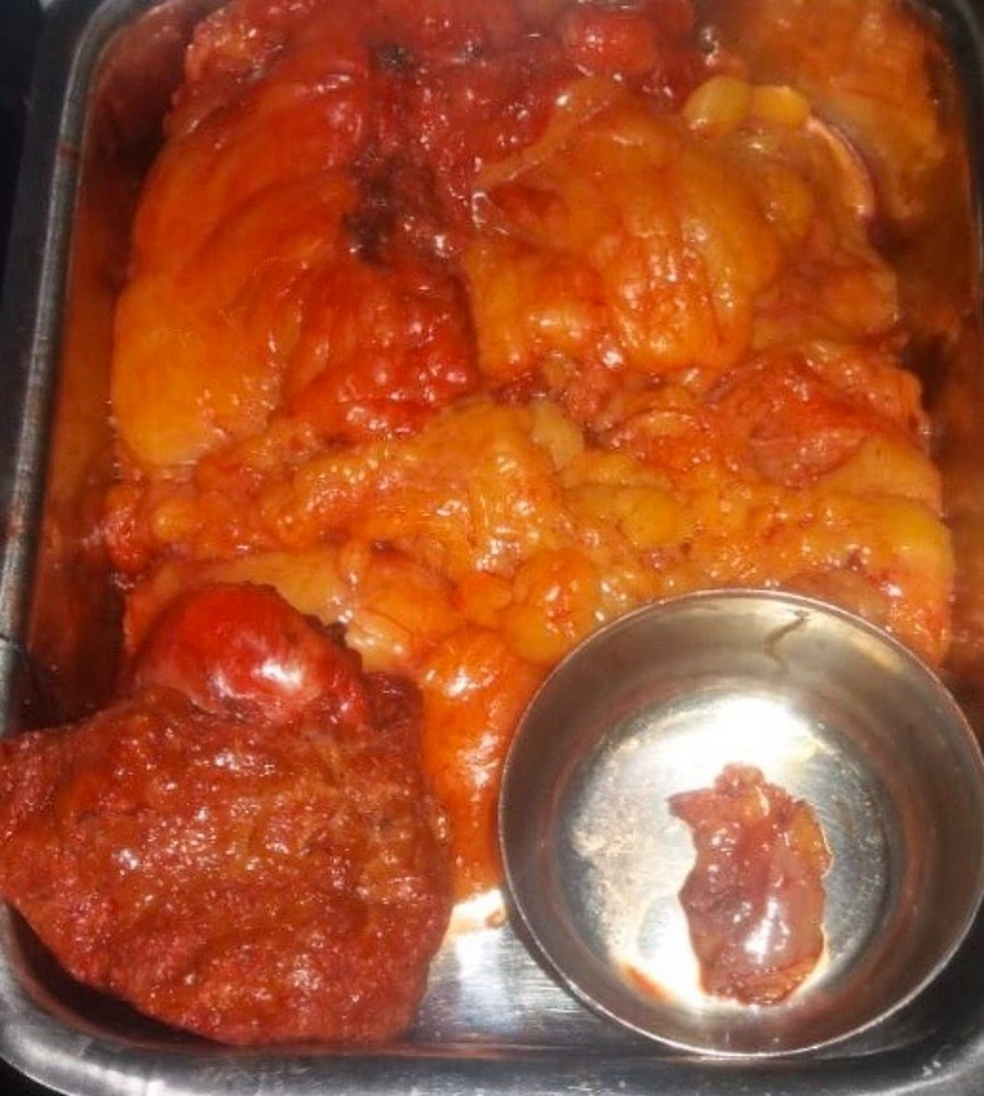
Resected specimen of radical cholecystectomy

She and her family were counseled regarding the available treatment options and prognosis. After taking informed consent, she received cisplatin + etoposide-based chemotherapy for three cycles. Repeat CECT and PET-CT showed shrinkage of the tumor suggesting a partial response to chemotherapy (Figure 4). She underwent open radical cholecystectomy after neoadjuvant chemotherapy. Resected specimens were sent for histopathological examination which confirmed the pre-surgery diagnosis (Figure 5). The postoperative period was uneventful and she was discharged on postoperative day 12. She received a six-week course of 54 Gy of radiotherapy with concurrent cisplatin and etoposide-based chemotherapy. She was advised to undergo ET scan yearly for the next three years to look for recurrence. Over a follow-up of 18 months, the patient is free of disease recurrence. 

## Discussion

Neuroendocrine tumors can occur anywhere in the body which is made up of enterochromaffin cells. The etiological identifiers of these tumors in the gallbladder are as follows: (1) The undifferentiated stem cells of the gallbladder separate into neuroendocrine cells; (2) the presence of cholelithiasis causes pathological gastric metaplasia due to chronic inflammation of the gallbladder mucosa. In an advanced state, this inflammation produces neuroendocrine cells, eventually leading to the development of a gallbladder NET [8].

These tumors of the extrahepatic bile ducts and the ampulla region are highly malignant due to poor differentiation and hence have a bad prognosis [9]. The common sites of metastasis are the liver, portal lymph nodes, lungs, and peritoneum [10]. Clinical presentations are nonspecific. A vague abdominal pain is often the most common symptom [11]. Few carcinoid symptoms were found, but they account for only 1% of all symptoms as most of these tumors are nonfunctional with no functional endocrine granules [12]. Radiological investigations such as USG, CECT can reveal gallbladder masses indicative of malignancy however it is impossible to preoperatively distinguish NET from other types of tumors. Histopathological and immunohistochemical staining such as synaptophysin and chromogranin are essential for diagnosis. These tumors invade into adjacent liver parenchyma and may cause intrahepatic biliary obstruction [13]. Due to the lack of early signs and the aggressive nature of the tumor, patients are diagnosed mostly at an advanced stage where lymphovascular invasion or liver parenchymal invasion had already taken place leading to a poor prognosis [14]. If the histopathological examination of the gallbladder specimen reveals involvement of only mucosal, submucosal, or muscular layer, simple cholecystectomy is adequate [15]. In locally advanced cases, radical cholecystectomy and lymphadenectomy combined with selective hepatic resections must be performed to achieve a tumor-free margin [14]. It is always recommended to perform radical resection to the maximum possible extent to improve the prognosis as the chance of recurrence in case of inadequate resection is very high. Volume reduction surgery for patients for whom radical resection is not possible improves the quality of life [16]. A better survival rate has been seen when surgical intervention is combined with adjuvant chemotherapy for locally invasive diseases. The most common chemotherapeutic drugs used in inoperable cases are streptozotocin, 5-fluorouracil, adriamycin, cisplatin, and etoposide and the choice of these drugs depend upon the differing degree of tumor differentiation [17]. With that said, no randomized, blinded trials have been performed to validate one regimen as the gold standard and treatment varies from person to person.

## Conclusions

Gallbladder neuroendocrine tumor is a very rare malignancy with an extremely poor prognosis. Due to its rarity, very few cases have been reported in the English medical literature. Early diagnosis and prompt surgical intervention can give the patient best outcome. Neoadjuvant and adjuvant chemotherapy provides a definable survival advantage to the patient but is without a well-defined standard of care protocol. Randomized, blinded trials have to be performed to validate one regimen of chemotherapy as the gold standard.
